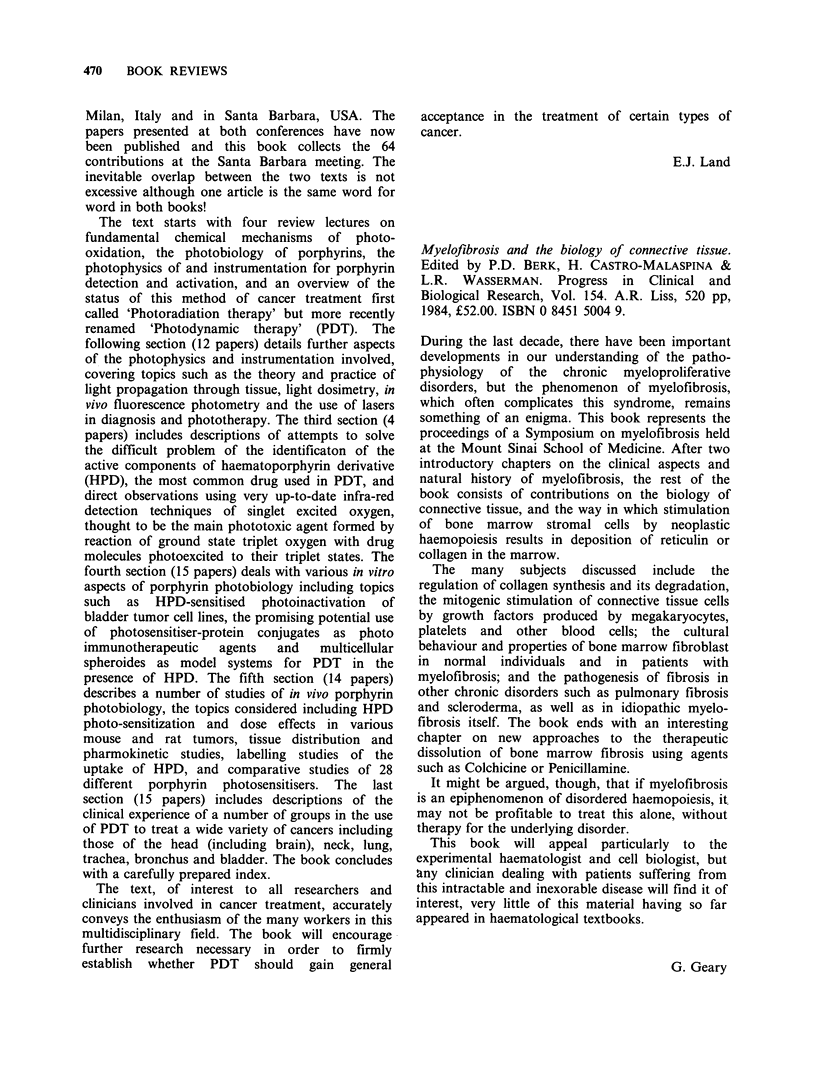# Myelofibrosis and the biology of connective tissue

**Published:** 1985-09

**Authors:** G. Geary


*Myelofibrosis and the biology of connective tissue.* Edited by P.D. BERK, H. CASTRO-MALASPINA & L.R. WASSERMAN. Progress in Clinical and Biological Research, Vol. 154. A.R. Liss, 520 pp, 1984, £52.00. ISBN 0 8451 5004 9.

During the last decade, there have been important developments in our understanding of the pathophysiology of the chronic myeloproliferative disorders, but the phenomenon of myelofibrosis, which often complicates this syndrome, remains something of an enigma. This book represents the proceedings of a Symposium on myelofibrosis held at the Mount Sinai School of Medicine. After two introductory chapters on the clinical aspects and natural history of myelofibrosis, the rest of the book consists of contributions on the biology of connective tissue, and the way in which stimulation of bone marrow stromal cells by neoplastic haemopoiesis results in deposition of reticulin or collagen in the marrow.

The many subjects discussed include the regulation of collagen synthesis and its degradation, the mitogenic stimulation of connective tissue cells by growth factors produced by megakaryocytes, platelets and other blood cells; the cultural behaviour and properties of bone marrow fibroblast in normal individuals and in patients with myelofibrosis; and the pathogenesis of fibrosis in other chronic disorders such as pulmonary fibrosis and scleroderma, as well as in idiopathic myelofibrosis itself. The book ends with an interesting chapter on new approaches to the therapeutic dissolution of bone marrow fibrosis using agents such as Colchicine or Penicillamine.

It might be argued, though, that if myelofibrosis is an epiphenomenon of disordered haemopoiesis, it may not be profitable to treat this alone, without therapy for the underlying disorder.

This book will appeal particularly to the experimental haematologist and cell biologist, but any clinician dealing with patients suffering from this intractable and inexorable disease will find it of interest, very little of this material having so far appeared in haematological textbooks.

G. Geary